# Building impactful systems-focused simulations: integrating change and project management frameworks into the pre-work phase

**DOI:** 10.1186/s41077-021-00169-x

**Published:** 2021-04-29

**Authors:** Mirette Dubé, Glenn Posner, Kimberly Stone, Marjorie White, Alyshah Kaba, Komal Bajaj, Adam Cheng, Vincent Grant, Simon Huang, Jennifer Reid

**Affiliations:** 1 eSIM Provincial Simulation Program, Alberta Health Services, Alberta, Canada; 2 Department of Community Health Sciences, Cumming School of Medicine, University of Calgary, Calgary, Canada; 3 Department of Obstetrics and Gynecology, University of Ottawa, Ottawa, Canada; 4 Department of Innovation in Medical Education, University of Ottawa, Ottawa, Canada; 5 The Ottawa Hospital, Ottawa, Canada; 6 University of Ottawa Skills and Simulation Centre, Ottawa, Canada; 7 Divison of Emergency Medicine, Department of Pediatrics, University of Washington School of Medicine, Seattle, USA; 8 Seattle Children’s Hospital, Seattle, Washington USA; 9 Division of Emergency Medicine, Department of Pediatrics, School of Medicine, University of Alabama at Birmingham, Birmingham, USA; 10 Department of Medical Education, School of Medicine, University of Alabama at Birmingham, Birmingham, USA; 11 Department of Health Services Administration, School of Health Professions, University of Alabama at Birmingham, Birmingham, USA; 12 Office of Interprofessional Simulation for Innovative Clinical Practice (OIPS), UAB Center for Interprofessional Education and Simulation (CIPES), Birmingham, USA; 13 UAB Clinical Simulation Program, UAB Health System, Birmingham, USA; 14 NYC Health + Hospitals/Jacobi, Bronx, New York, USA; 15 Albert Einsten College of Medicine, Bronx, USA; 16 Department of Pediatrics and Emergency Medicine, Cumming School of Medicine, University of Calgary, Calgary, Canada; 17 KidSIM, Alberta Children’s Hospital, Calgary, Canada; 18 Alberta Children’s Hospital, Calgary, Canada; 19 Department of Emergency Medicine, Queen Elizabeth II Health Sciences Center, Dalhousie University, Halifax, Canada

**Keywords:** Simulation, Systems integration, Quality improvement, System improvement, Patient safety, Change management, Project management, Systems simulation, Systems-focused debriefing

## Abstract

Healthcare organizations strive to deliver safe, high-quality, efficient care. These complex systems frequently harbor gaps, which if unmitigated, could result in harm. Systems-focused simulation (SFS) projects, which include systems-focused debriefing (SFD), if well designed and executed, can proactively and comprehensively identify gaps and test and improve systems, enabling institutions to improve safety and quality before patients and staff are placed at risk.

The previously published systems-focused debriefing framework, Promoting Excellence and Reflective Learning in Simulation (PEARLS) for Systems Integration (PSI), describes a systematic approach to SFD. It includes an essential “pre-work” phase, encompassing evidence-informed steps that lead up to a SFD. Despite inclusion in the PSI framework, a detailed description of the pre-work phase, and how each component facilitates change management, was limited.

The goal of this paper is to elucidate the PSI “Pre-work” phase, everything leading up to the systems-focused simulation and debriefing. It describes how the integration of project and change management principles ensures that a comprehensive collection of safety and quality issues are reliably identified and captured.

## Background

Systems issues, inefficiencies, and latent safety threats lurk in modern healthcare organizations. Previous publications have described the use of simulation and debriefing to identify safety threats and inefficiencies during design [1, 2] prior to opening [3] and after opening [4] new clinical units and/or implementing new processes [5].

Patient safety science promotes proactive identification of systems issues to mitigate harm, before patients are placed at risk. Systems-focused simulation (SFS) coupled with systems-focused debriefing (SFD) is an emerging quality improvement tool [6]. During SFS, both routine and high-risk situations are simulated, using real equipment, team members, environments, and processes. SFS and SFD facilitate the identification of safety threats, inefficiencies, and opportunities for quality improvement at all levels of the system, without placing patients at risk. Furthermore, SFS and SFD can aid in highlighting and reinforcing system resilience and organizational learning from simulation.

When issue identification during a SFS is integrated with an evidence-informed approach to mitigation, latent safety threats can be proactively mitigated [7]. To maximize the impact of SFS and SFD, a systematic approach to the pre-work, built on the principles of project and change management, with attention from intake to execution, can ensure comprehensive, effective interventions [8]. Without investing adequately in the “Pre-Work” phase, there is a risk that SFS/SFD will fail to discover important latent safety threats and/or create lasting change.

The PEARLS-PSI approach is grounded in interdependent project and change management principles. Multiple frameworks exist for project management [9–12]. Project management methodologies describe key phases of a project (e.g. initiation, planning, execution, monitoring, controlling and closing), while offering specific perspectives and tools to ensure success [10, 11]. The Project Management Body of Knowledge (PMBOK) developed by the American National Standards Institute and global organizations such as the International Organization for Standardization (ISO), describes standard terminology, guidelines and best practices that can serve as a foundation for any project management methodology [12, 13]. By incorporating best practices for project management into each phase of Pre-work for SFS/SFD, important steps are reliably addressed, and project structure standardized.

**Fig. 1:**
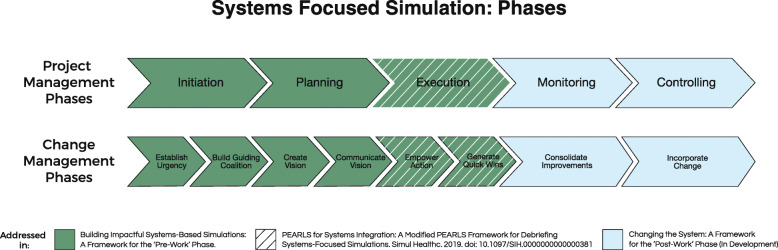
Systems-focused simulations: phases. Providing an overview of systems simulation with project and change management phases

Change management frameworks guide how we prepare, support, and equip team members to successfully catalyze change [14]. Multiple frameworks exist for change management, with organizations varying in their adoption. What is important for SFS/SFD is that change principles are methodically utilized to purposefully build agency in teams and promote lasting change. A generally recognized model, developed by Dr. John Kotter [15], describes eight essential elements that contribute to creating lasting change. The pre-work phase of PSI integrates Kotter’s first six steps (Fig. 1). At the onset, opportunities are identified, goals clarified, and a timeframe established. This develops a key principle, creating a sense of urgency. Subsequently, in the pre-work phase, a core stakeholder group is assembled, with the expertise and power to lead, working together to identify key concerns. They ensure that together, a representative and comprehensive vision is crafted. This encompasses two key change principles: build a guiding coalition and create a shared vision. Once the vision of the SFS/SFD has been created, a critical step in the pre-work phase, and in change management, is to communicate that vision to a wider range of stakeholders. This larger “army,” including participants, observers, and issue mitigators, can align resources to remove obstacles to change. At the end of the pre-work phase, planning for, creating, and communicating short-term wins can help catalyze the change continuum. By incorporating these change principles into the pre-work phase of SFS/SFD, the likelihood of a successful, sustainable impact for SFS/SFD reliably increases.

There is limited guidance published on the preparatory steps required for SFS and SFD [16] that methodically integrates project phases and change management principles. The PEARLS for Systems Integration (PSI) publication focuses on a systems-focused debriefing framework that includes tools and scripts for an SFD [17]. It does not describe critical components leading up to the SFD itself. The purpose of this paper is to describe in detail the pre-work phase of PSI, including project initiation, planning, and execution: all the elements that lead up to a SFS/SFD, including preparation of participants and space for the SFS/SFD itself. We have included “how to” tools, to assist simulationists in identifying key components and interdependencies.

**Fig. 2:**
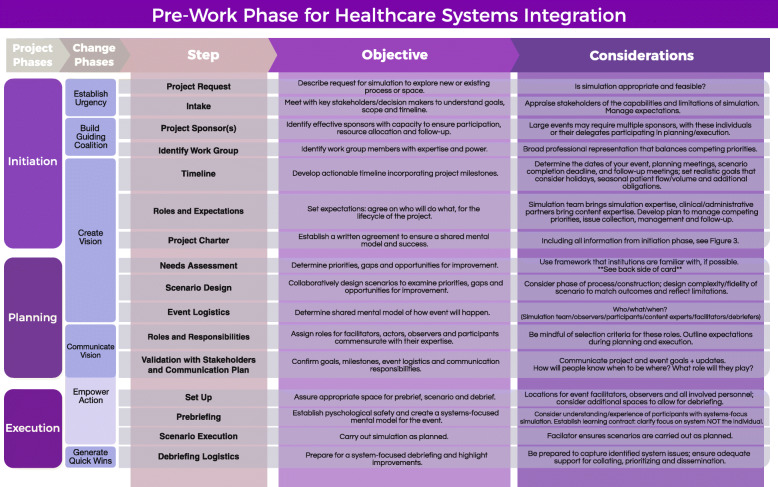
Pre-work phase for healthcare systems integration. Listing all steps within a project approach including objectives and considerations for simulation for systems integration

Figure 2 outlines key steps in the pre-work phase, identifies how they relate to project and change management, and includes specific objectives and considerations. These steps can be applied to any SFS project focused on environmental and/or process changes.

Throughout this manuscript, we will explore an example: a new neurology clinic. The new clinic is located within a larger hospital. It is expanding the number of exam rooms from 4 to 10, including 2 rooms for video EEG studies, a new outpatient service. The increase in clinic size and services includes the addition of 2 new providers and 6 new nurses, medical assistants, and techs. The clinic sees up to 40 patients/day, including up to 2 video EEG studies/day. Patients include those at risk for seizures, particularly in the EEG studies, who occasionally need emergency assistance with their breathing. New technology, including alarms and intercoms, was installed to help staff recruit help more easily. A new role, a medical assistant, was added. New processes, including an emergency response plan, were developed. New environmental changes include the new location, rooms, and set-up for emergency equipment in each exam room.

## SFS project initiation

The main objective of initiation is to define the project, creating a shared mental model between the SFS/SFD team and stakeholders.

### Project request

Requests may come from anyone looking to design, test, or improve a space or process. The first step is determining suitability of SFS/SFD: feasibility factors include organizational priorities and culture, safety objectives, program goals, stakeholder engagement, preparedness, timelines, and available resources.

### Initial intake

Identify critical stakeholders. Without them, important information, informing underlying system issues and root causes, may be missed. Consider including administrative leaders, educators, and clinical and non-clinical leaders.

Individuals with a background in SFS/SFD should describe the difference between systems-focused simulation and learner-focused simulation for the planning team, as this difference may not be common knowledge. By the end of the intake process, the project team should have agreement on sponsors, timelines, and roles, and start a simulation event charter, which establishes a shared sense of urgency.

In our example, critical stakeholders in the neurology clinic include clinical leadership representing frontline staff: EEG technicians, nurses, neurology providers, medical assistants, and receptionists. Additional stakeholders include the hospital medical response team, adjacent clinic leadership, who also respond to emergencies, and the emergency department, who would receive any unstable patients. Non-clinical stakeholders include engineering, who oversees functionality of the new alarms.

### Simulation event project charter

**Fig. 3:**
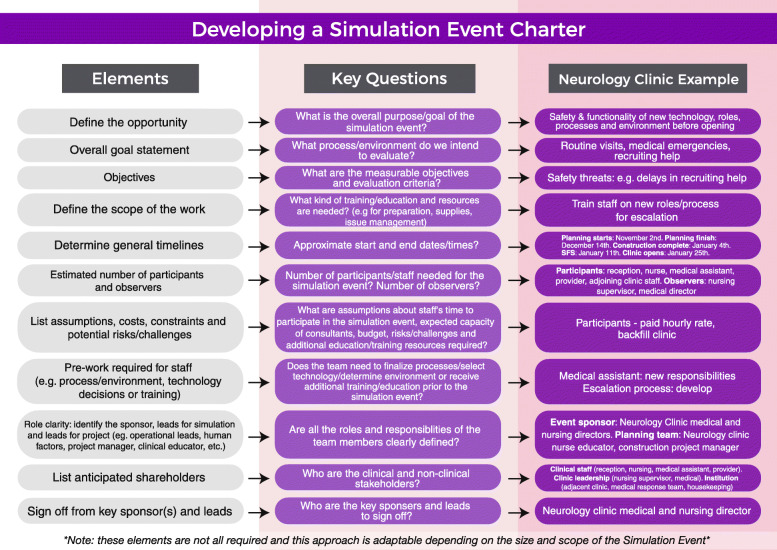
Developing a simulation event charter. The elements and key questions to answer in a systems simulation event charter with an applied example

In SFS/SFD, the project charter, a foundational project management tool, establishes agreements and assures their delivery [10]. Without a charter, the risk of misunderstandings between stakeholders relative to timelines, objectives, and who is involved is higher and may threaten a project’s success. It may be a stand-alone document (Fig. 3) or be included as part of a systems-simulation scenario template, depending on scope and amount of information to document. Key elements include goals, timelines, and responsibilities agreed upon in the initiation phase.

### Timeline

Concrete timelines should be determined collectively with all stakeholders. Identify milestones and document these in the event charter (e.g., completion dates for objectives, scenario development, when the space/process will be close to “patient ready,” systems-focused simulation event, and follow-up meetings). Project management experience indicates that lack of established timelines may result in resistance, failure to produce deliverables, and extended timelines [10].

For the neurology clinic example, stakeholders agreed on a 13-week timeframe from the initial stakeholder meeting to the projected clinic opening, including 2 weeks to mitigate systems issues and accommodate re-testing, if required, after the SFS/SFD. Simulation planning team members delegated additional staff for set-up of the new space and staff training. The neurology clinic was the only clinic opening, not part of a multi-clinic opening. Resources such as information technology, facilities, and clinical engineering could focus on mitigating issues specifically for the neurology clinic during the 2 weeks prior to opening. Considerations which could extend the project timeline include work loads of planning team members or key stakeholders, training needs or resources, and focused resources for issue mitigation.

### Project roles and expectations

Clarify project team roles and expectations for each stage of the project: initiation, planning, execution, and follow-up. For complex projects, different “working groups” may be responsible for different phases of the project (e.g., scenario design, logistics, mitigation plans, and/or communication). These roles contribute to the guiding coalition and ensure progression through different phases of change.

An important change management step involves assigning senior member(s) of the team who are able to address resistance and build change competency within the organization [14, 18]. For SFS projects, the sponsor is identified early, communicates directly with key stakeholders and participants, and attends initial planning meetings, remaining closely connected with the planning team. The sponsor(s) may be called upon to approve resources, such as paid staff project time, supplies, or equipment. Sponsors leverage influence to involve resistant stakeholders, promote issue mitigation, and support implementation.

Stakeholders prioritize issues, develop recommendations, assign operational owners, and allocate resources. SFS/SFD consultants partner with designated content experts from each stakeholder group to plan the SFS/SFD according to the agreed upon objectives and timelines. Unless SFS/SFD consultants have additional quality improvement roles, they might not work on mitigating identified systems issues.

For our neurology clinic, the medical and nursing director served as sponsors. The nursing manager and nurse educator partnered with the construction project manager and simulation consultant to form the core planning team, as they had deep understanding of changes in the new clinic and potential impact on the clinical team. Additional stakeholders included providers, medical assistants, techs, engineers, and equipment supply chain. Representatives for each stakeholder group validated SFS plans and provided resources, as needed.

## SFS project planning

### Needs assessment

**Fig. 4:**
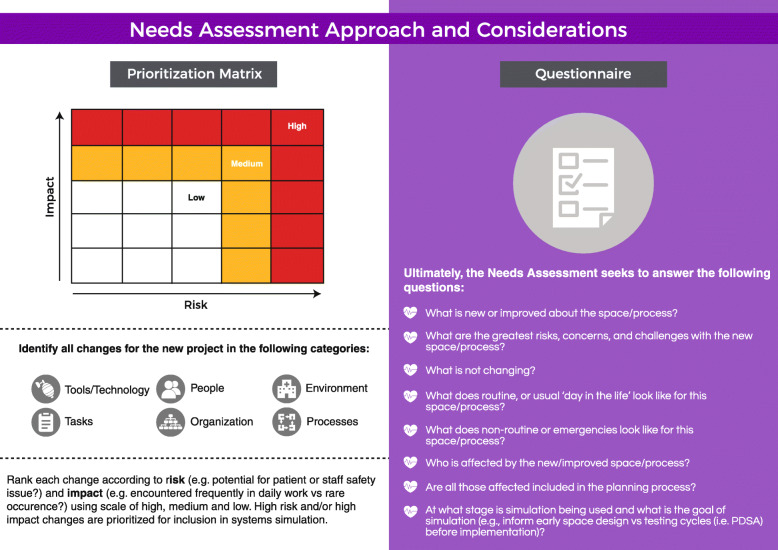
Needs assessment approach and considerations. Using a needs assessment approach including a commonly used prioritization matrix and/or questionnaire

A formal needs assessment, implemented at the onset of planning, informs objectives of the SFS/SFD, identifying gaps and opportunities for improvement. Figure 4 provides a tool for conducting a needs assessment, including key questions and a prioritization matrix to ensure that the highest risk and highest impact objectives are prioritized.

To apply a prioritization matrix, the stakeholder group identifies all changes within the project scope (e.g., tools, technology, roles, environment, processes) associated with the new space/process, then ranks each change according to risk and impact. With impact defined as the frequency of occurrence. The highest risk and/or highest impact changes are prioritized for inclusion in a systems simulation(s). This approach is based on the Failure Mode and Effects analysis (FMEA) [19], a tool gaining popularity for use with both planning and evaluation of issues identified in SFS/SFD. Focusing on high-risk/impact changes for inclusion in SFS/SFD facilitates teams directing their improvement work to areas with the greatest patient safety impact [20, 21]. The prioritization matrix described here has been modified from the traditional FMEA format, to decrease complexity and improve ease of application for stakeholders. Rather than including specific risk profiling numbers, the concept is addressed more generally, assigning global categories of high, medium, and low for both risk and frequency. Changes identified as high risk or high frequency, similar to those with high RPN in a traditional FMEA, become objectives for the SFS/SFD. Specific failure modes cause and effects can then be examined and explored in the SFS/SFD. Outcomes or solutions for each objective are then based on observation rather than supposition, as in the traditional FMEA. Logistically, needs assessments may be conducted via a facilitated discussion, by utilizing audience response systems or asynchronously.

See Table 1 for an example of needs assessment and prioritization matrix for the neurology clinic example

**Table 1:** Needs assessment—neurology clinic example

Step 1: Planning group identifies changes (objectives) in each category	Step 2: Rank according to impact/risk	Step 3: Build scenarios incorporating identified changes (objectives)
**Example:**	**Example:**	**Example:**
**New neurology clinic**	**Impact:** frequent event =high impact, intermittent frequency= medium impact, rare event= low impact	**Scenario A:** Routine patient visit for seizures. Patient sees neurologist, gets EEG, has seizure, requires emergency response and transfer
	**Risk:** If fails, risk for significant patient harm= high risk. If fails, possibility of minor patient harm/delays in care that is likely to be detected = medium risk. If fails, unlikely to result in harm/delays and likely to be caught= low risk.	**Scenario B:** Routine visit for chronic headaches.
**Objectives**	**Impact, risk**	**Scenario**
**People (roles/responsibilities):**		
New medical assistant role	High impact, low risk	A +B
Adjacent clinic nursing staff will respond to emergencies in neurology clinic	Low impact, high risk	**A**
**Processes:**		
New process for rooming patients	High impact, low risk	A + B
New process for communicating EEG reviews	High impact, medium risk	A
New response team/process for emergency response	Low impact, high risk	A
**Tools/technology:**		
New phones	High impact, medium risk	A + B
New alarm system	Low impact, high risk	A
**Environment:**		
New waiting area to be shared with other clinics	High impact, low risk	A + B
New emergency equipment layout	High impact, high risk	A
New EEG room, distant from clinic/team	Medium impact, high risk	A
New transport route to emergency department	Low impact, high risk	A

### Scenario design

Scenario design translates the priorities identified in the needs assessment into scenario objectives (Table 1), incorporating best practices in scenario design [22]. If stakeholder groups are missing from scenario design and/or the SFS/SFD, scenarios may fail to accurately recreate complex systems and identify potential systems issues.

SFS scenarios focus on the elements needed to recreate functional spaces or process, rather than details related to closing individual knowledge gaps. For our neurology example, an objective of the scenario is to examine the functionality of a new patient room, both for routine EEG patients and those needing emergency care. Debriefing points focus on tools, tasks, teams, environment, and process, e.g., how space/tools worked for the triage through to assessment workflow, routine patient assessment, and assisting with a breathing emergency. The quality of the medical or nursing assessment is neither an objective nor a debriefing point.

When designing the scenario, give specific attention to identifying participants, necessary embedded participants, actors, or desired observers. Consider including participants less intimately involved in designing the new space/process to provide more objective feedback about “how the new space really works” vs. “how it should work” [4, 23]. Consider including participants or observers who can serve as trainers/superusers of the new space/process, leveraging the SFS as opportunity to create deep understanding about both the benefits and challenges of the new space/process.

If including patient and family representatives, ensure they are pre-briefed on the scenario content and pre-determined objectives to prevent situations which may be intellectually or emotionally difficult.

Larger systems-based simulations often draw a crowd of observers, which has potential to overwhelm the participants who may feel like they are “on stage”. Consider the physical space available, required professional perspectives, who may be responsible for ongoing training, and observer roles, who may have expertise in capturing specific objectives. Some observers may be selected to capture specific metrics or for areas of expertise. Video capture/streaming can also be used to broadcast the SFS/SFD to a separate space for observers, reducing the number of people in the simulation space. Reiterating a clear focus on the system, and not individual performance, throughout the pre-work phase is key.

### SFS event logistics

Simulation event logistics include detailed planning for the location, timing, and ownership of each component of the SFS/SFD. Detailed planning empowers “an army” to act on the SFS vision.

#### Location

Venue is dictated by objectives of the project and scenarios. For the new neurology clinic, the reception area was included to test patient flow from arrival to provider visit, plus clinic rooms and hallways connected to adjoining clinics to test a medical emergency and ability of support teams to respond. Locations may be limited by active construction or actual patient care needs. Consider the impact of busier/quieter times of day and higher/lower staffing on the objectives, scenarios, and effects on on-going operations at your venue. Identify specific spaces for pre-briefing, training participants and observers, debriefing, event coordinator command center, data collection, and an “off-stage” area for participants to re-group. A successful event accounts for smooth transition of all participants/activities with minimal disruption to ongoing patient care or functions.

#### Equipment/supplies

Creating a realistic space/process requires equipment, technology, and supplies. Ideally, all equipment would be “real”—those that will be used when the new space/process goes live. Practically, this is not always possible, as some equipment may have yet to arrive, be in use, or be cost prohibitive. Review each item for operational impact of using real vs. simulated items, assigning responsibility for procurement.

For our neurology clinic example, testing the functionality of the emergency equipment was a high priority. Clinic staff obtained real equipment, e.g., bag-mask, suction supplies, monitoring cables, from the current clinic to include in the SFS and ensured the new alarms would be fully functional prior to simulation. EEG machines were still in use in the current clinic; stakeholders identified that their biggest concern was about the location of EEG machine in the room and the impact of wires connecting the patient to the machine on an emergency response. The EEG machine was simulated using a box, roughly the same size, with strings attached to represent the wires.

### Day of event: roles and responsibilities

Table 2 outlines the key simulation event roles and their responsibilities.

**Table 2:** Simulation event roles and responsibilities

Roles	Responsibilities	Selection criteria for roles
**Event coordinator (may be a simulation team member)**	Primary oversight for the event. Directs and monitors flow. Determines detail decisions, solutions to problems during the event. Assigns, monitors, and supports facilitators for each component of the prebriefing, SFS, and SFD. Assign check in responsibilities (i.e., sign in, name tags, food, other set up), issue management, and communication responsibilities. Identify if and assign simulation technicians to provide simulation equipment and assist with logistics.	Experience in leading SFS/SFD. Project management training is an asset to this role.
**Simulation team**	Support the event coordinator. Operationalize the SFS/SFD by directing, facilitating, prebriefing, debriefing, operating any manikins/simulation equipment, and assisting in issue capture, as directed by the event coordinator and SFS/SFD plan.	Experience in SFS/SFD
**Patient/family partners/representatives (or actors)**	“Road test” the new space/process, add realism, provide unique point of view in the SFS/SFD	Identify individuals who may have been involved in the design process. *Note: Review SFS content prior to the event to ensure emotional compatibility*
**Observers**	Identify and record systems issues.	Select members of the working group(s) and supporting leadership positions (operational, educational, etc.) who can witness and compare “how work is being done” to the proposed design.
**Participants**	Perform their actual role. “Road test” the new space/process.	Select individuals not intimately involved in the design process to ensure objectivity and identify individuals who can serve as trainers for others after the event
**Administrative assistants**	Assists with logistics and recording/collating systems issues	Strong organization skills and attention to detail

### Validation and communication plan

At the conclusion of the initiation and planning phases, validation is a checkpoint to ensure that the shared vision is communicated broadly and has been operationalized, or if not, determine what steps are needed.

Validation can take different forms. For larger simulation events, such as those involving multiple spaces/teams/processes, stakeholders may meet in person. Collectively, they review the charter, needs assessment, scenario plans, and resources, ensuring critical roles/considerations have not been omitted and that resources are committed. For smaller events, validation may be accomplished electronically or in sequential discussions. Incorporate time to discuss differences of opinion and concerns.

Communication serves as the backbone for change, empowering others to act and share short-term wins. Create a structure for relaying event plans, invitations, and instructions.

## Execution

The execution phase, a standard project management phase, includes elements of the pre-work phase, such as selection and preparation of orientation spaces and pre-briefing modules for participants, as well as the SFS/SFD itself. Pre-work includes all set-up and preparation of the participants and observers, prior to starting the simulation scenarios and debriefing.

### Set-Up

Identify adequate spaces for pre-briefing, training, and event coordination. Pre-briefing space should include check-in, event role assignments, consent, name tags, and visual cues, e.g., different colored vests based on roles. Designate space for pre-briefing the group as a whole, as well as any subgroup training needed for participants, family representatives, observers, etc. Identify a coordination center, where the event leader can be easily accessed, administrative support can collate issues and resource representatives, such as clinical engineering or vendors, can be found for problem-solving.

To set up for SFD, consider both smaller, scenario-specific debriefings distributed throughout the event, as well as larger debriefings. Space and technology options may vary. Safety issues/themes identified could be collected on white boards, large post-it notes, paper logs, or computerized records. Regardless of method, a complete record of all systems issues will help make issues and improvements visible, reinforcing short-term wins and preparing for more change following the SFS.

For our neurology clinic example, the clinic reception area served for check in, pre-briefing, and large group debriefing. Subgroup training took place in both the reception area and nursing station. An office adjacent to the nursing station served as the event coordination center, allowing event coordinators and resource representatives to be physically located near the center of scenarios.

### Prebriefing

Prebriefing establishes the purpose of an SFS/SFD, to identify systems and safety issues for the purpose of systems improvements. Much like validation, when the vision of the event is communicated to a wider range of stakeholders during the planning phase, the prebrief communicates the vision to all those involved in the execution phase. This is particularly important if participants are accustomed to learner-focused simulations where the focus is on individual knowledge, teamwork, and skills [17]. In contrast with learner-focused simulation, specific goals, such as functionality of emergency equipment and activation of an emergency team, are usually shared prior to the scenario. SFS usually benefit from “full disclosure,” sharing anticipated medical/behavioral decision making to ensure objectives are met. This includes informing participants and observers of the types of scenarios to be included: routine patient visit, medical emergency secondary to a seizure, etc. In addition, critical actions needed to test the system, such as using emergency equipment, activating alarms, and integrating emergency response teams, should be disclosed. Disclosure serves two purposes: to reduce participants’ anxiety about feeling “tested” in their decision-making and to ensure that the system is fully tested. A detailed agenda and goals of the event are shared with all participants and observers, a technique that helps ensure focus remains on pertinent systems issues.

Prebriefing should include an orientation to the new space/process, event agenda, event roles, and any event-specific expectations. Recognition of performance improvements and key employees previously involved during the planning phase shares short-term wins and helps set the stage for the future change and needs. Include a pre-established process should a real emergency occur, such as the use of a key phrase like “time-out” or “no duff” to encourage participants and observers to clarify concerns and promote safety.

### Scenario execution

Following through on thoughtful initiation and planning phases, the scenario execution finally allows the team to conduct the scenario as designed. The event leader ensures that the plan is followed and/or decides on necessary modifications to meet objectives. Plan for timely communication amongst the event/simulation team should scenarios or SFD(s) take more or less time than anticipated, if multiple scenarios need to be coordinated or unanticipated barriers arise. Consider group texting, walkie talkies, or institutional or private phones. Specific scenario facilitators, as well as embedded participants, may guide participants based on the pre-established scenarios and objectives. For our neurology clinic example, two scenario facilitators conducted scenarios simultaneously. A group text gave each of them the ability to update the others on scenario progress and to cue an embedded participant to begin having a seizure.

### Debriefing logistics

Depending on event size, number, and progression of scenarios, there may be one or multiple debriefings. Some events benefit from multiple focused SFDs, after critical phases in each scenario, to address each objective, which may be followed by a meta-debrief to share themes across scenarios. For the neurology clinic, focused debriefings occurred at critical junctures in each scenario. A family representative and receptionist are debriefed at the end of the check-in process. A nurse and family representative are debriefed after the rooming and initial assessment process. Many team members were debriefed after providing airway assistance when the patient had a seizure. Large group debriefing, including all participants, highlighted observations from the focused debriefings. If observer and participant numbers become too large, time is limited, or there are objectives focused on specific groups, consider hosting separate debriefing events.

Large post-it notes or projected live capture on a screen can be used to log systems issues into organized categories [16, 17, 24]. Consider sorting issues into broad categories, such as processes, environment, and tools, or tailoring them for specific event goals, such as emergency equipment layout, to prompt more feedback. For large events, designated scribes may record systems issues, while the lead facilitator guides discussion and summarizes key systems issues.

Planning for a feedback mechanism for individual participants, either electronic or paper, allows for additional data collection, particularly those less willing to share in groups.

At the conclusion of the pre-work phase, each element of the systems-focused simulation and debriefing should have been planned for, set up, and ready to go. The pre-work typically takes the longest, encompassing work by many stakeholders and team members. When done systematically and thoroughly, incorporating project and change management principles, the SFS and SFD proceed as designed with the greatest chance of achieving their goals: the identification of safety and quality issues and improving systems integration.

## Summary

The pre-work phase of planning for an SFS/SFD project is informed by combining project management phases and change principles with system-focused simulation methodologies to provide an evidence-based, standardized approach. This approach guides healthcare organizations to successfully identify, capture, and improve process and systems issues using simulation. Considering and incorporating each step (Fig. 2) of the initiation, planning, and execution of a system simulation project will help ensure quality outcomes and create lasting change. Embedding change management principles into SFS design prepares teams for the post-work phase, increasing the likelihood that new systems and processes are institutionalized and serve as the catalyst for more improvement. Proactively and maximally improving patient safety, enabling system improvements, and promoting organizational learning are the ultimate goal of any systems-focused simulation and debriefing event.

## Data Availability

Data sharing is not applicable to this article as no datasets were generated or analyzed.

## Abbreviations

IT: Information technology
FMEA: Failure modes and effects analysis
PEARLS: Promoting Excellence and Reflective Learning in Simulation
PSI: PEARLS for Systems Integration
RPN: Risk profile numbers
SFS: Systems-focused simulation
SFD: Systems-focused debriefing
